# Sliding Inguinal Bladder Hernia: An Open and Minimally Invasive Robotic-Assisted Repair

**DOI:** 10.7759/cureus.35207

**Published:** 2023-02-20

**Authors:** Andrei Gritsiuta, Makayla Gologram, Christopher Myers, Christopher Esper

**Affiliations:** 1 Surgical Services, University of Pittsburgh Medical Center, Pittsburgh, USA; 2 Plastic Surgery, Lake Erie College of Osteopathic Medicine, Erie, USA

**Keywords:** sliding hernia, minimally invasive surgical procedures, robotic surgical procedures, bladder hernia, inguinal hernia repair

## Abstract

Inguinal hernia repair, although a common procedure, can present in complicated ways such as a sliding inguinal bladder hernia (IBH). This rare type of hernia can alter a patient’s quality of life by obstructing urination, requiring manual scrotal compression to fully empty the bladder, and lead to devastating complications such as hydronephrosis and kidney failure. Treatment is typically by open inguinal hernia repair with manual bladder reduction, but this method poses risks of iatrogenic injury to the bladder. Within this case series, IBH repairs via open and robotic-assisted laparoscopic procedures are compared, and the morbidity and mortality of each method are analyzed. Although risk of recurrence is similar for both procedures, robotic surgeries are linked to decreased postoperative pain and length of hospital stay. The ease of dissection of pelvic anatomy and detailed view of the associated structures that robotic surgery can provide during a complex hernia repair encourages its use for IBH.

## Introduction

Inguinal hernia repair is one of the most common procedures performed by general surgeons in the United States. Although most elective presentations are routine, the inguinal hernia can present in a more complicated manner such as sliding, where an organ and its overlying peritoneum will develop a wall of the hernia sac, possibly containing retroperitoneal mesentery. Sliding inguinal bladder hernias (IBH) constitute less than 4% of all inguinal hernias, typically found in men older than 50 and with comorbid obesity, bladder outlet obstruction, and prostatic hypertrophy [1-3]. While many small IBH are asymptomatic, larger hernias can have significant symptoms including difficulty urinating, dysuria, scrotal edema, and even severe hydronephrosis or renal failure [4,5]. Some patients require manual scrotal compression to urinate and facilitate complete bladder emptying which can cause significant impairment to a patient’s quality of life [6]. Surgical repair is necessary to reduce IBH and, while minimally invasive approaches have recently been explored, open repair has typically been preferred using the Lichtenstein technique [7]. The current recommendations and guidelines for this repair do not suggest that one technique, open, laparoscopic, or robotic, to be superior than one another [1-12]. With the increased use of robotic technology throughout the country, the goal of this case series is to describe our experience with open and robotic-assisted laparoscopic surgical repair of a IBH while encouraging the meticulous dissection capabilities of robotic surgery to favor its use for these complex hernias.

## Case presentation

Patient 1: Open approach

**Figure 1:**
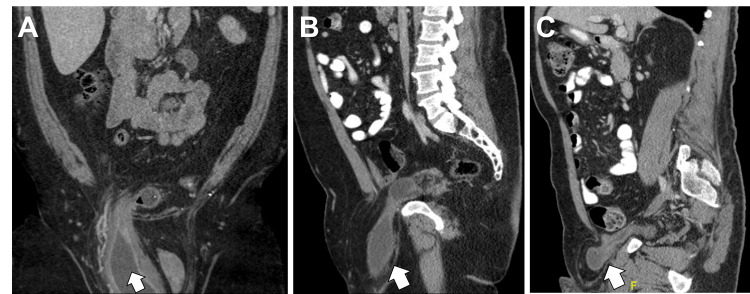
Preoperative CT images. Images of sliding inguinal bladder hernia (white arrows) of (A) a 72-year-old male (coronal view); (B) a 46-year-old male, and (C) a 68-year-old male (sagittal views).

A 72-year-old Caucasian male (BMI of 42.9 kg/m^2^) with a past medical history significant for a suprapubic catheter placement secondary to neurogenic bladder and high-pressure urinary retention presented with progressively enlarging right groin bulge, scrotal swelling, and decreased urine output. He followed with urology for frequent cystoscopies, multiple urinary tract infections with multi-resistant *Escherichia coli* and monthly catheter changes. Prior to presentation, the patient had to squeeze the right side of his scrotum to produce urine form his suprapubic catheter and that urine output recently became bloody, prompting him to present to the emergency department. Physical examination revealed a large, nonreducible, right-sided inguinal hernia, nontender on palpation, and on attempted reduction urine flow was produced from the suprapubic catheter. Computed tomography (CT) showed a right inguinal hernia sac, measuring more than 14 cm in diameter craniocaudally, containing the entire bladder with an enhancing rim and thickened wall measuring as much as 13.5 mm, and a loop of small bowel abutting the proximal aspect of the hernia (Figure 1A).

**Figure 2:**
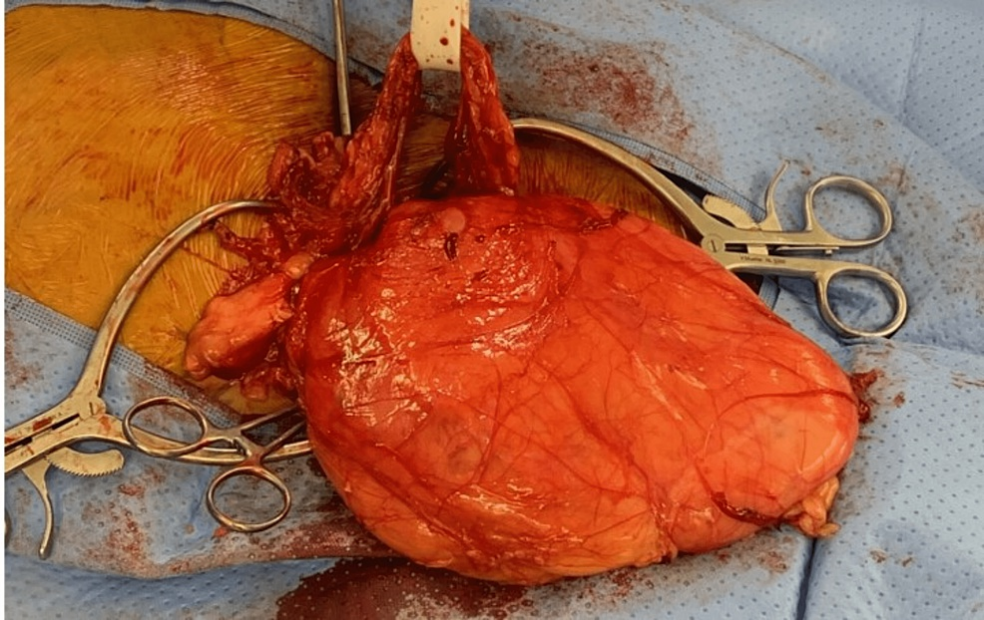
Intraoperative view of inguinal bladder hernia. The cord contents were able to be separated from the hernia sac and protected with Penrose drain.

Distal portions of the ureters, bilaterally, appeared to be involved in the neck of the hernia and a large 9.3 x 7.8 cm fluid collection with a fat component in the right hemiscrotum was found compressing the left hemiscrotum. After a multidisciplinary evaluation, the patient was taken to the operating room for open inguinal hernia repair with mesh. A very large, direct sliding-type defect involving the wall of the bladder was identified (Figure 2).

The bladder was drained via the suprapubic catheter allowing for easier manipulation of the hernia sac. The cord structures were able to be separated from the hernia sac, they were stretched quite thin but appeared to be intact. The entirety of the hernia was reduced with the assistance of the Trendelenburg position and the direct M3F0 defect (>3 cm without signs of femoral hernia according to European Hernia Society (EHS) classification) was primarily reconstructed by Lichtenstein technique using ProGrip 10 x 15 cm mesh (Covidien, Mansfield, USA). A Foley catheter was placed via the urethra at the conclusion of the procedure which produced thick purulent urine and was sent for culture and speciation. Operative time was 105 minutes with an estimated blood loss (EBL) of 50 ml. Postoperatively, the patient was found to have positive urine cultures for *Klebsiella* and *Group B Streptococcus*, which required a prolonged course of antibiotics. After an uncomplicated hospital stay, he was discharged in stable condition on a third postoperative day (POD). The Foley catheter was removed two weeks postoperatively by urology. The patient remained asymptomatic at a 12-month follow-up visit with no indication of hernia recurrence.

Patients 2 and 3: Robotic-assisted laparoscopic approach

Two patients with IBH underwent robotic-assisted laparoscopic repair with mesh via the transabdominal preperitoneal (TAPP) approach using the Da Vinci Xi Robotic System (Intuitive Surgical Inc., Sunnyvale, CA). A 46-year-old Caucasian male with past surgical history significant for open left inguinal hernia repair with mesh presented with a three-week history of worsening intermittent pain in the right groin. He had also noted a mass in this region, but the physical examination was complicated by obese body habitus (BMI of 39.49 kg/m^2^) and fat deposition. CT scan revealed a large, right inguinal hernia containing abdominal fat and most of the urinary bladder (Figure 1B). After weight reduction and smoking cessation, the patient was scheduled for an elective right inguinal hernia repair. The patient was positioned in Trendelenburg and a Foley catheter was placed to decompress the bladder. Using standard port site placement for an inguinal hernia repair, the robotic system was docked and the pelvis was explored. The large right indirect inguinal hernia was identified containing the entire urinary bladder. No hernias in the left groin. And abnormal mass was found to be attached to the fat surrounding the sigmoid colon. The mass was freed from surrounding fat, stapled off, and sent to pathology for a frozen section. The pathology diagnosed fat necrosis. Given this benign finding, dissection of the hernia was resumed. There was no injury identified to the bladder, and after complete dissection of the right groin, a ProGrip 15 x 9 cm mesh (Covidien, Mansfield, USA) was used to cover the L3Fx defect. The Foley catheter was removed at the end of the case. The patient tolerated the procedure well without postoperative complications and was discharged on POD#2.

**Figure 3:**
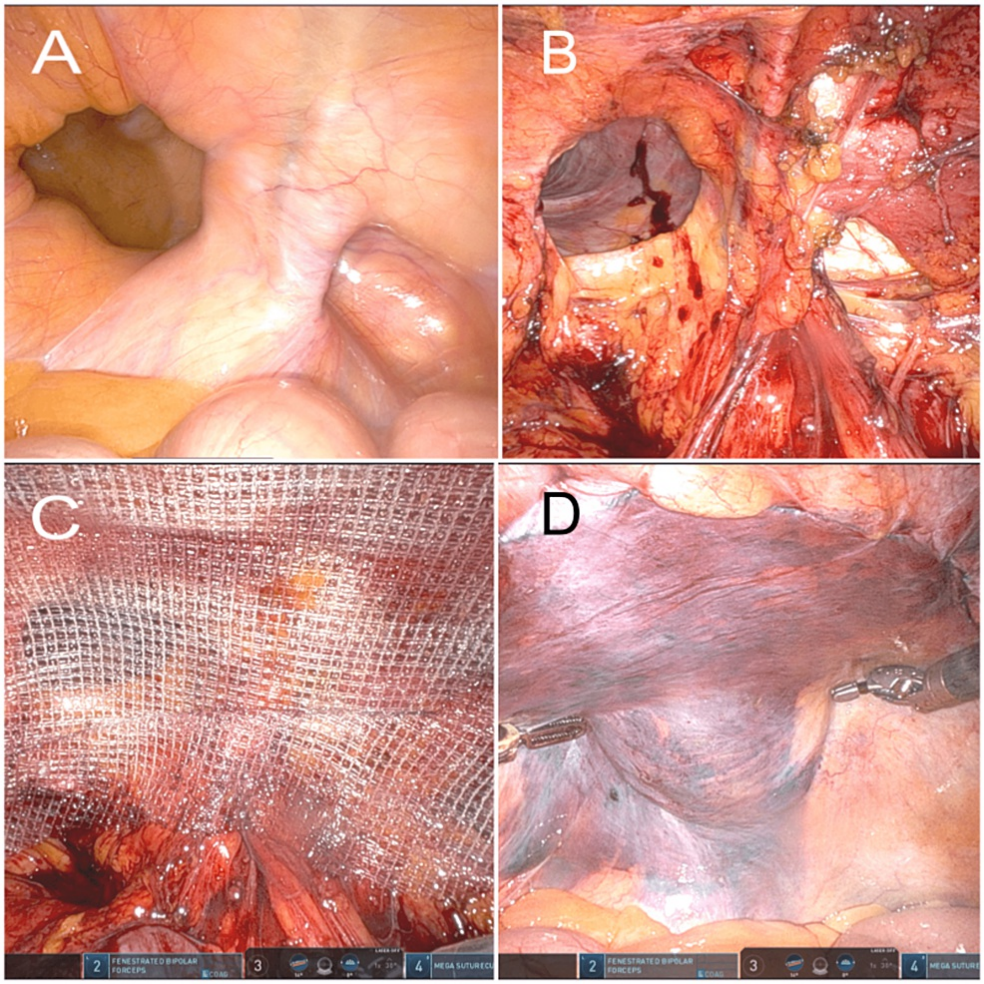
Intraoperative images. (A) pantaloon right inguinal hernia after reduction; (B) preperitoneal dissection; (C) hernia defect is repaired with ProGrip mesh; (D) peritoneal closure using V-Loc suture.

Another 68-year-old Caucasian male (BMI of 25.5 kg/m^2^) presented with asymptomatic umbilical and bilateral inguinal hernias. CT scan showed bilateral inguinal hernias containing fat with the right inguinal hernia containing a portion of the right anterior wall of the urinary bladder with focal dilation of the distal portions of both ureters just proximal to the ureterovesical junction (Figure 1C). Intraoperatively, a pantaloon hernia with the bladder as a portion of the medial direct hernia sac was identified (Figure 3A), as well as a contralateral direct hernia defect.

Bilateral hernia defects were repaired in a similar TAPP fashion using ProGrip 15 x 9 cm mesh. After pain control, the patient was discharged on the same POD without postoperative complications.

In both cases, large inguinal hernia defects were readily recognized, containing the urinary bladder. The bladder was carefully mobilized from dense adhesions and completely reduced to the abdominal cavity without injury. The total duration of surgery for unilateral and bilateral repairs was 363 min and 130 min, respectively, with minimal EBL. Prolonged operative time in the first case was related to unexpected intraoperative findings and awaiting frozen pathology results which were unrelated to the hernia repair and found to be benign. Both patients remained asymptomatic at a three-month follow-up and had no evidence of hernia recurrence.

## Discussion

A sliding hernia is described as the herniation of a retroperitoneal organ through the abdominal wall such as the cecum, ascending colon, sigmoid colon, ureters, and bladder in a male patient. These hernias can be present as unilateral or bilateral, direct, or indirect. IBH is rare, making up 4% of sliding hernias [7]. Diagnosing these typically requires CT imaging although some may be found incidentally during inguinal hernia repair. Sliding IBH has a higher rate of recurrence and/or reoperation than other inguinal hernias and the chosen surgical repair should take this matter into consideration [8].

The complexity of IBH as well as the difficult anatomy of the pelvis and inguinal region makes any surgical approach challenging. Open repair of these hernias is the most common approach to management with 80.4% of cases being done open [7]. Preoperative diagnosis of these hernias decreases the risk of intraoperative complications, but rates of bladder injury can be as high as 12% [6]. Open repairs using the Lichtenstein technique have once been the preferred technique. The size of the hernia defect and any concomitant bladder ischemia or necrosis could complicate a minimally invasive approach and increase rates of conversion to open, but ultimately the surgeon’s techniques and control of a robotic-assisted technique will dictate its use, particularly for complicated sliding inguinal hernias. Robotic-assisted laparoscopic surgery improves dexterity and visualization of the associated pelvic anatomy, potentially reducing iatrogenic risk to the bladder, which may outweigh the longer operative times and higher surgical costs linked to this practice. Three traditional port site locations were used for the robotic approach whereas open repairs may use a large incision above the inguinal ligament or a Pfannenstiel incision to access the herniated bladder [9]. Minimally invasive surgery was found to have a greater visualization of sliding hernia and a lower risk of recurrence when compared to open [10]. The open repair compared to a robotic approach potentially increases postoperative pain, length of stay, and risk of complications such as partial cystectomy. Failure to recognize bladder involvement can result in significant postoperative morbidities such as gross hematuria, fistula formation, infection, and sepsis [9]. Although laparoscopic surgery was not performed in this study, the approach is similar to that of the robotic approach and the same repair, TAPP, was used.

Benefits to the robotic approach seen in the case series include a less invasive entrance to the defect and decreased postoperative pain and length of hospital stay. There is minimal literature written on the laparoscopic and robotic repair of IBH. Open repairs have been the standard of care for these hernias, but the strong preference for robotic inguinal hernia repairs by experienced minimally invasive surgeons may encourage this approach. The TAPP repair used in these cases proved excellent dissection margins with no evidence of iatrogenic injury to the bladder or other surrounding anatomy. TAPP repairs for inguinal hernias have low to no risk of recurrence up to four years after repair and minimal rates of any complication. When operating on complex hernias with complicated anatomy, it’s important to use a surgical approach that can guarantee a detailed anatomical view and aid in the fine dissection of delicate pelvic structures, encouraging a robotic-assisted laparoscopic approach to be the preferred method for sliding bladder hernias.

## Conclusions

Within this case series, IBH repairs via open and robotic-assisted laparoscopic procedures are discussed. Although the risk of recurrence is similar for both procedures, robotic surgeries are linked to decreased postoperative pain and length of hospital stay. The ease of dissection of pelvic anatomy and detailed view of the associated structures that robotic surgery can provide during a complex hernia repair encourages its use for IBH.
